# Supplementary material for: Organophosphate Pesticides and Pyrethroids in Farmland of the Pearl River Delta, China: Regional Residue, Distributions and Risks
Source: Int J Environ Res Public Health. 2023 Jan 5;20(2):1017. doi: 10.3390/ijerph20021017 (PMC9858657; doi:10.3390/ijerph20021017)
Supplement: Supplementary file 1 [file ijerph-20-01017-s001.zip › ijerph-2127637-supplementary.pdf]

Supporting Information

Organophosphate Pesticides and Pyrethroids in Farmland of the Pearl River Delta, China: Regional Residue, Distributions and Risks

Runlin Yao [1,2], Siyu Yao [3], Tao Ai [1,*], Jiahui Huang [1], Yang Liu [1] and Jianteng Sun [1,*]

1 Guangdong Provincial Key Laboratory of Petrochemical Pollution Processes and Control, School of Environmental Science and Engineering, Guangdong University of Petrochemical Technology, Maoming 525000, China
2 Bathurst Future Agri-Tech Institute, Qingdao Agricultural University, Qingdao 266109, China
3 Department of Environmental Sciences, College of Earth and Environment Sciences, Lanzhou University, Lanzhou 730000, China
* Correspondence: aitao@gdupt.edu.cn (T.A.); sunjianteng@zju.edu.cn (J.S.)

2.5. Health and Ecological Risk Evaluation

The exposure non-cancer risk of OPPs was calculated with the methods recommended by the US EPA [1] to evaluate the health risk of OPPs to inhabitants. Two primary routes, including soil ingestion and dermal contact, were applied to assess the human health risk. The average daily doses (ADD, mg kg^{-1} day^{-1}) of soil ingestion and dermal contact exposure routes were calculated as follows in the assessment.

$$ADDingest=\frac{Csoil\times IRS\times CF\times EF\times ED}{BW\times AT} \quad (1)$$

$$ADDdermal=\frac{Csoil\times CF\times SA\times AF\times ABS\times EF\times ED}{BW\times AT} \quad (2)$$

where Csoil means the concentration of OPPs in agricultural soil (mg·kg^{-1}); IRS is the agricultural soil ingestion rate (mg·day^{-1}); EF is the exposure frequency (days $year^{-1}$); ED is the exposure duration (years); BW is the body weight (kg); AT is the average lifetime exposure (days); SA is the dermal surface area (cm^2·day); AF is the soil adherence factor (mg·cm^2); ABS is the fraction absorbed dermally from soils (unitless); CF is the conversion factor (kg·mg^{-1}). The non-cancer risks of OPPs via soil ingestion and dermal contact were presented as HI, which was calculated in the following equations:

$$HQ=\frac{ADD}{RfD} \quad (3)$$

$$HI=\sum HQ_i=\sum\frac{ADD_i}{RfD_i} \quad (4)$$

Where HQ is defined as the hazard quotient; RfD (mg·kg^{-1}·day^{-1}) represents the daily maximum permissible level of OPPs, including the reference dose for ingestion (RfD_0) and the reference dose for dermal contact (RfD_{ABS} = RfD_0 ×ABS_{GI}). ABS_{GI} is the fraction of OPPs sorbed in gastrointestinal tract (unitless); i means the different exposure pathways.

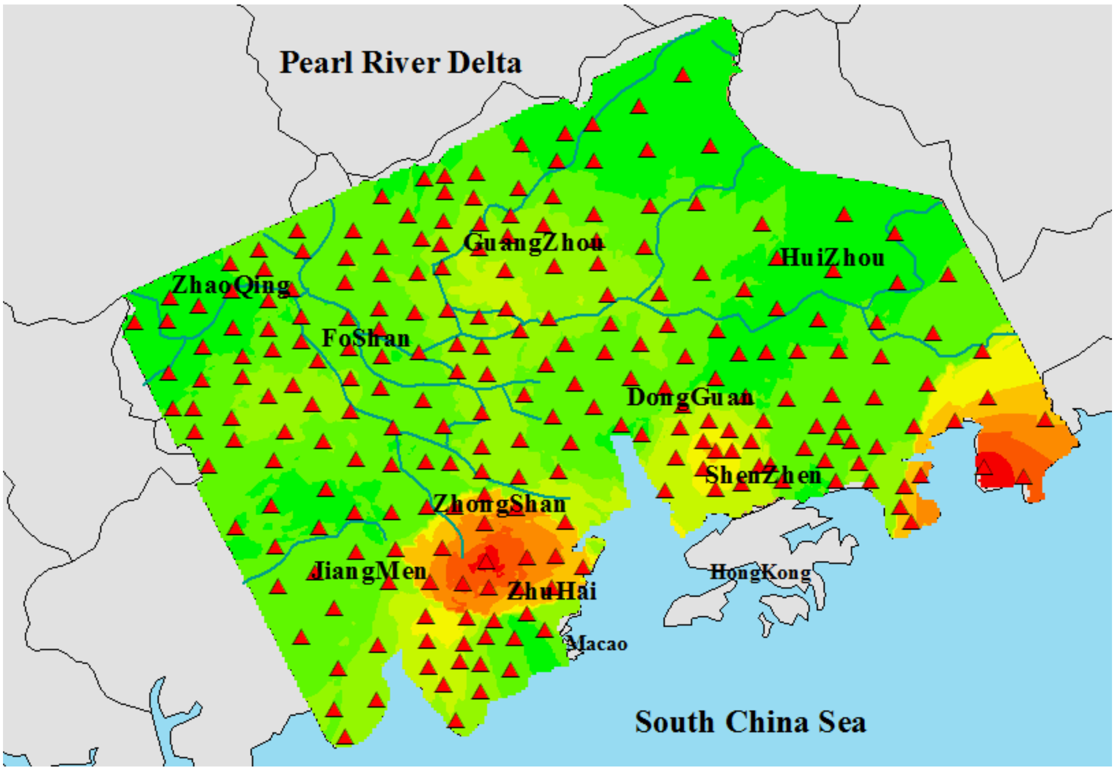

Figure S1. The sampling sites in the Pearl River Delta region.

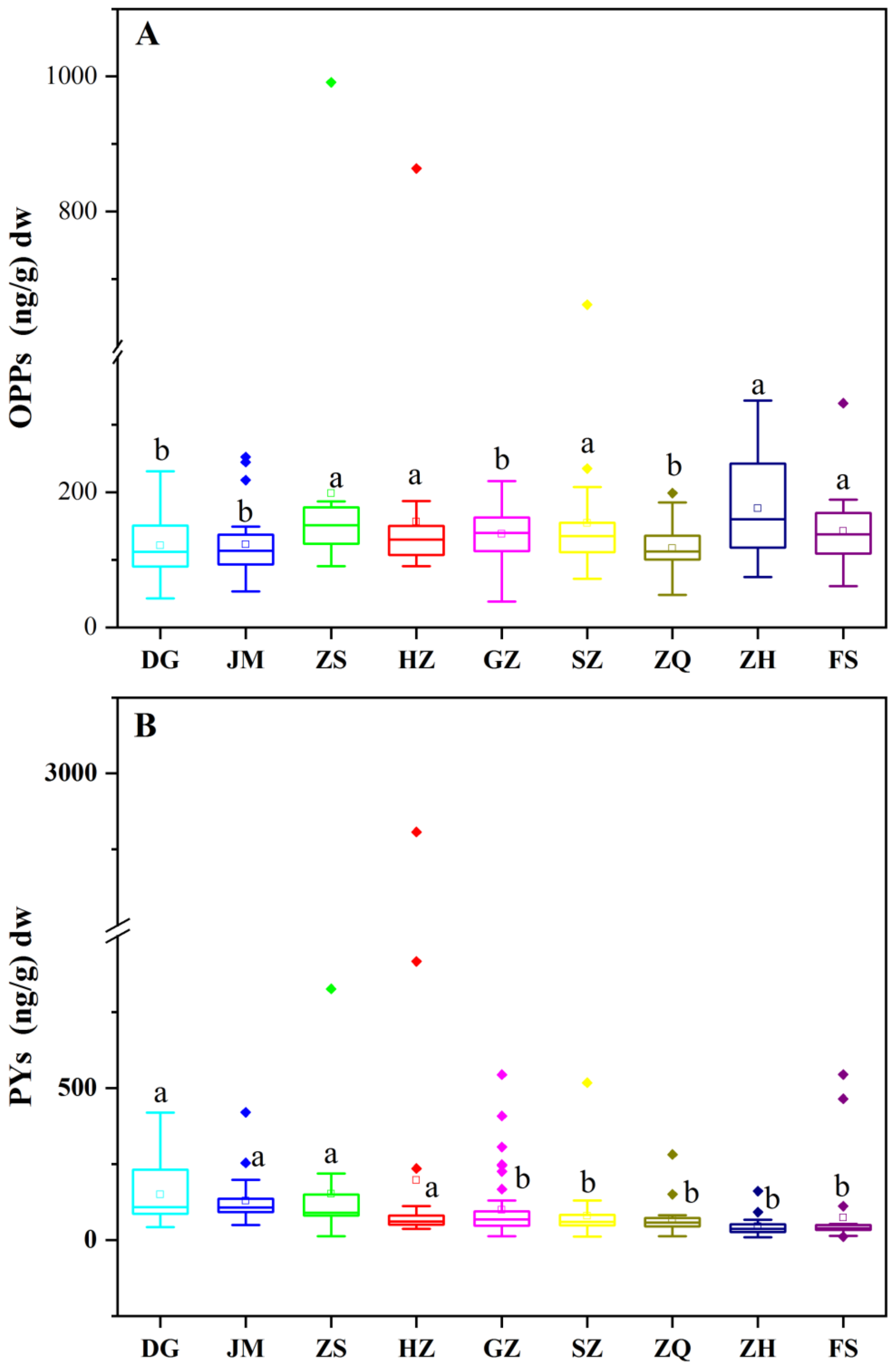

Figure S2. The total concentration of the OPPs (**A**) and PYs (**B**) in different cities in the agricultural soils of the PRD. Different letters (a and b) indicate the significant differences among treatments ($p < 0.05$, one-way ANOVA followed by LSD test).

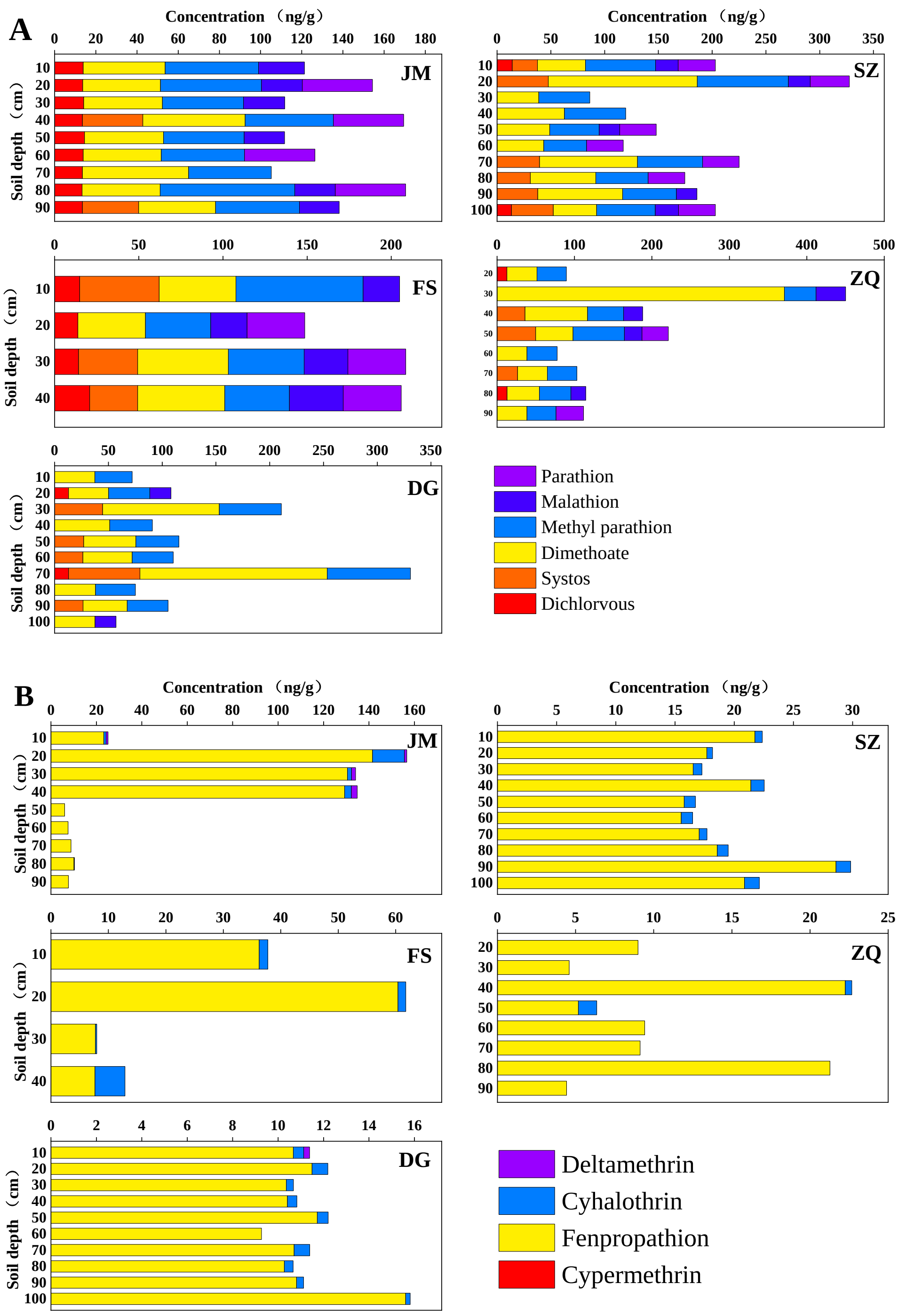

Figure S3. Vertical distributions of the concentrations and compositions of OPPs (**A**) and PYs (**B**) in agricultural soils of PRD region.

.

Table S1. Average recovery, RSD, and limit of detection (LOD) for the analytical method of pesticides in soil.

Compounds	Recovery (%)	RSD	LOD (ng/g)
Dichlorvos	82	4.41	4.6
Demeton	76	3.91	5.5
Dimethoate	89	1.73	3.0
Malathion	86	5.31	3.1
Parathion	97	3.93	3.2
Methyl parathion	87	4.41	4.1
Cypermethrin	82	7.6	0.2
Fenpropathion	99	9.1	0.4
Cyhalothoin	91	8.5	0.1
Deltamethrin	101	8.1	0.5

Table S2. Parameters for adults and children in exposure risk assessment (US EPA. 2015).

Parameter			Value
IRS	Adults		100
	Children		200
EF			350
ED	Adults		26
	Children		6
BW	Adults		80
	Children		15
AT	Adults		9490 (non-cancer)
			25550 (cancer)
	Children		2190 (non-cancer)
			25550 (cancer)
SA	Adults		6032
	Children		2373
AF	Adults		0.07
	Children		0.2
ABS			0.1
CF			10^{-6}
RfD_0			0.035
ABS_{GI}			1

References

1. USEPA. *Mid-Atlantic RISK Assessment*; Untied States Environmental Protection Agency: Washington, DC, USA, 2015.
